# Iranian neonatal diabetes mellitus due to mutation in *PDX1* gene: a case report

**DOI:** 10.1186/s13256-019-2149-x

**Published:** 2019-08-01

**Authors:** Leyla Sahebi, Nikoo Niknafs, Hosein Dalili, Elahe Amini, Tahereh Esmaeilnia, Mahsa Amoli, Nahid Farrokhzad

**Affiliations:** 1 Institute of Family Health , Breastfeeding Research Center, Tehran University of Medical Science, Tehran, Iran; 2 Institute of Family Health, Maternal-Fetal and Neonatal Research Center, Tehran University of Medical Science, Tehran, Iran; 3 Metabolic Disorders Research Center, Endocrinology and Metabolism Molecular-Cellular Sciences Institute, Tehran University of Medical Sciences, Tehran, Iran

**Keywords:** Neonatal diabetes mellitus, *PDX1* gene, Mutation

## Abstract

**Background:**

Neonatal diabetes mellitus with hyperglycemia during the first 6 months of life is a rare disorder that can occur in all races and societies.

**Case presentation:**

In this study, we introduced an Iranian (Persian) 65-day-old patient with neonatal diabetes mellitus with novel homozygous mutation in the pancreatic and duodenal homeobox 1, *PDX1,* gene, which is also known as *IPF1* gene, located in exon 2. This case was a newborn boy born in Vali-Asr Hospital, Tehran; he was diagnosed as having hyperglycemia on 28th day. Genetic analysis detected a homozygous mutation on *PDX1* gene on chromosome 13. It is a novel homozygous mutation in the *PDX1* gene (NM_000209.3), p.Phe167Val. This mutation was confirmed by Sanger sequencing. There was no evidence of agenesis of the pancreas.

**Conclusions:**

We reported a case of neonatal diabetes mellitus due to novel homozygous mutation in the *PDX1* gene without exocrine pancreas manifestations.

**Electronic supplementary material:**

The online version of this article (https://doi.org/10.1186/s13256-019-2149-x) contains supplementary material, which is available to authorized users.

## Introduction

Neonatal diabetes mellitus (NDM) is a monogenic form of diabetes [1] that is characterized by hyperglycemia and the need for insulin treatment within the first 6 months of life [2]. Symptoms of NDM include frequent urination and dehydration. NDM can be diagnosed by finding elevated levels of glucose in blood or urine. In severe cases, the deficiency of insulin may cause the body to produce an excess of acid, resulting in a potentially life-threatening condition called ketoacidosis [3]. NDM is a rare condition occurring in only one in 100,000 to 500,000 live births [3].

Clinically, NDM can be divided into three subgroups: (i) transient NDM (TNDM) in which insulin secretion is spontaneously recovered by several months of age (at a median age of 3 month); (ii) permanent NDM (PNDM) requiring lifelong medication; and (iii) PNDM existing as part of a syndrome (syndromic NDM). This phenotypic classification is useful, as the most common causative genetic abnormalities differ by each subtype, although overlap exists [1, 4].

Recent molecular analysis of NDM identified at least 12 responsible genes: chromosome 6q24, *KCNJ11*, *ABCC8*, *INS*, *FOXP3*, *GCK*, *IPF1* which is also known as pancreatic and duodenal homeobox 1 (*PDX1*), *PTF1A*, *EIF2AK3*, *GLUT2*, *HNF1β*, and *GLIS3* [4]. Defects on chromosome 6q24 (approximately 70%) and the *KCNJ11* mutation have been recognized as the major causes of TNDM and PNDM, respectively, in Caucasians imprinting [1]. Early diagnosis as well as early insulin therapy initiation prevents the metabolic catastrophe of ketoacidosis and development of chronic and irreversible complications. Determination of clinical and molecular characteristics of this disorder helps to manage the short-term and long-term treatment, and, finally, it provides new achievements for future research. In this study, for the first time, we reported the case of an Iranian patient with NDM with novel homozygous mutation in *PDX1* (*IPF1*) gene located in exon 2.

## Case presentation

### Clinical manifestations

Our patient was born by caesarean section in Vali-Asr Hospital, Tehran. He was an Iranian 65-day-old boy and was the second child of consanguineous healthy parents. It is noteworthy that his mother had a history of gestational diabetes and his parents’ relatives had diabetes mellitus type 2 history too (second-degree relatives). He was born at 38 weeks of gestation with birth weight of 1800 g (< third percentile) and length 46 cm (< third percentile). His blood pressure was 57–89.7 mmHg with failure to thrive (FTT). This newborn had normal anterior fontanelle, soft abdomen, and undescended testis. Other clinical examinations revealed heart rate (HR) 190 beats per minute, Saturation of Peripheral Oxygen (spO_2_) 5 minutes 87%, without respiratory distress syndrome (RDS).

### Laboratory diagnostic methods

A drop of capillary blood (drawn from the heel) was sampled and applied to a test strip (Gluco Easy; Kyunggi, South Korea) to measure level of glucose. Before sampling, the baby’s heel was warmed up by hand massage followed by disinfecting the spot, and then a blood sample was taken.

Two ml of arterial blood was taken by a trained nurse. White blood cell (WBC) were counted with automated counters by Wright or May–Grünwald–Giemsa technique. Hemoglobin (HGB) and platelet (PLT) determinations were performed by an automated cell counter too. C-reactive protein (CRP) was measured in ethylenediaminetetraacetic acid (EDTA)-blood samples by a rapid immunometric method. A standard enzymatic test was used to measure alanine aminotransferase (ALT) and aspartate aminotransferase (AST). Bilirubin was measured by chromatography and amylase was measured by photometry (α-Amylase Kit).

### Laboratory and imaging findings

An ultrasound of our patient revealed duodenal atresia, fatty liver, and normal spleen and pancreas. Echocardiography showed atrial septal defects (ASDs). Ultrasound of his brain revealed germinal matrix hemorrhage (GMH); electroencephalography was abnormal.

A magnetic resonance imaging (MRI) study demonstrated hypogenesis of the corpus callosum. A lumbar puncture culture was negative.

Blood examination on the first day (day of birth) showed disseminated intravascular coagulation (DIC), anemia (Hb, 6/4), metabolic acidosis, thrombocytopenia (PLT, 116), hyperbilirubinemia, and neonatal cholestasis. The laboratory findings after the first 24 hours of birth are presented in Table 1.

**Table 1:** The clinical report, treatment process, and laboratory findings after the first 24 hours of birth

Neonate’s age	Clinical report	Treatment progress	Laboratory findings								
			HB (g/dL)	WBC (n/ml)	CRP (mg/l)	BS (mg/dl)	Bili-T (mg/dl)	Bili-D (mg/dl)	Amylase	AST (U/L)	ALT (U/L)
One-day-old to 7-day-old	-Severe IUGR -Embryonic ultrasound document = duodenal atresia detection -HR = 130 -RR = 40 -spO_2_ = 95% -Echocardiography = ASD	-Admission in NICU -Oxygen therapy by oxygen hood -Broad-spectrum antibiotic therapy (ciprofloxacin, colistin, linezolid, amphotericin B) -Injection of pack cell, IVIG, fresh frozen plasma, cryoprecipitate, and G-CSF -Surgery of duodenal atresia	6.4	2.9	–	–	–	–	–	–	–
7-day-old to 20-day-old	-BP = 75/43(mmHg) -Ultrasound of kidneys = normal Ultrasound of liver = normal Ultrasound of brain = GMH -Electroencephalography = Abnormal	-Phenobarbital; 3 mg/kg	10.5	4.49	–	–	–	–	–	–	–
20-day-old to 25-day-old	-Lumbar puncture culture = negative -Glucose test every 2 hours = hyperglycemia	Subcutaneous insulin injection/0.03 unit	–	–	–	-First three levels every 2 hours were: 280, 300, 496 -After subcutaneous regular insulin injection: 360	–	–	–	–	–
26-day-old	-spO_2_ = 98% -BP = 92/48 -Glucose test every 2 hours = hyperglycemia -MRI = hypogenesis of the corpus callosum	Subcutaneous regular insulin injection; 0.03unit	8.7	–	–	157	–	–	–	–	–
26-day-old to 31-day-old	-Glucose test every 2 hours -Stool exam = acholic Stool -Rubella IgG: high -CMV IgG: high -PCR CMV = negative	-Subcutaneous regular insulin injection; 0.03 -	9.3	5.4	68.0	206	–	–	–	–	–
32-day-old	-BP = 87/72 -Glucose test every four hours -Diarrhea Resistance to insulin Pancreas ultrasound = normal	-Intravenous insulin injection; if patient is NPO (start the dose of 0.02 unit)	–	–	–	200	4.4	3.9	2.9	57	42
32-day-old to 40-day-old	-Genetic counseling -Pedigree determination -Genotyping	-Probability of neonate diabetes	–	–	–	–	–	–	–	–	–
40-day-old to 48-day-old	Glucose test every 4 hours Stool exam = acholic Stool -Fisting in fingers -Severe FTT -Doppler ultrasound = fatty liver -Eye examination = normal	- Intravenous insulin injection (0.1 u/Kg/hour; if BS > 250) Glibenclamide prescription -Ursobil (ursodeoxycholic acid) prescription	4.6	34.0	–	–	–	–	–	–	–
48-day-old to 53-day-old	-Glucose test every 4 hours	-If BS > 250 prescription insulin, if BS < 50 prescription dextrose 10%	–	–	–	–	–	–	–	–	–
57-day-old	-Glucose test every 4 hours	-Regular insulin;0.2 and NPH insulin; 0.4 after 48 hours				-After regular insulin; 0.2: 564 After NPH insulin; 0.4: 228					
60-day-old	Glucose test every 4 hours	-If BS > 250 prescription insulin, if BS < 50 prescription dextrose 10%				166					
65-day-old	Discharged at 65-days old against medical advice										

ALT alanine aminotransferase, ASD atrial septal defect, AST aspartate aminotransferase, Bili-D bilirubin direct, Bili-T total bilirubin, BP blood pressure, BS blood sugar, CMV cytomegalovirus, CRP C-reactive protein, FTT FailureTo Thrive, G-CSF granulocyte colony-stimulating factor, GMH germinal matrix hemorrhage, HGB hemoglobin, HR heart rate, IUGR intrauterine growth retardation, IVIG intravenous immunoglobulin, MRI magnetic resonance imaging, NICU neonatal intensive care unit, NPH isophane insulin, NPO Nothing by Mouth, PCR polymerase chain reaction, RR respiratory rate, spO_2_ Saturation of Peripheral Oxygen, WBC white blood cell

### Genetic findings

**Fig. 1:**
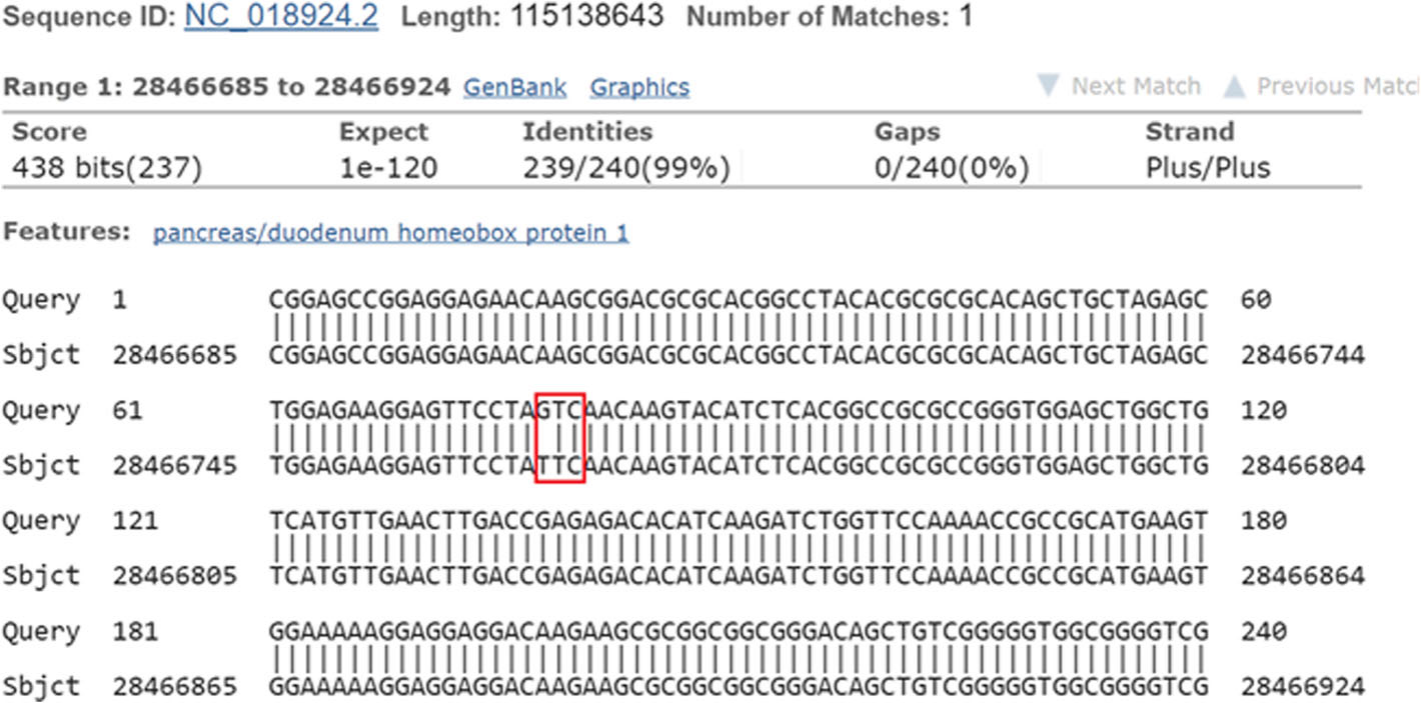
The sequenced data showing mutation in c.499 T>G of *PDX1* gene

Genetic analysis detected a homozygous mutation on *PDX1* gene on chromosome 13: 27,920,020-27,926,231 (c.499 T>G) (Fig. 1) (Additional file 1: Figures S1 and S2). (GRCh38): 13:27,919,981-27,926,313 with cytogenetic location: 13q12.2 and genomic coordinates (GRCh38): 13:27,919,981-27,926,313.

This transcript has two exons, is annotated with 18 domains and features, and associated with 214 variations and maps to 38 oligo probes. This gene is a member of the human consensus coding sequence (CCDS) set. It is a novel homozygous mutation in the *PDX1* (*IPF1*) gene located in exon 2 (NM_000209.3), p.Phe167Val. This mutation was confirmed by Sanger sequencing. Some *PDX1* mutations and their phenotypes shown in Table 2.

**Table 2:** Some *PDX1* mutations and their phenotypes

Phenotype	Mutation	dbSNP
Pancreatic agenesis 1 maturity-onset diabetes of the young, Type 4, included	*PDX1*, 1-BP DEL, 188C	–
Diabetes mellitus, Type Ii, susceptibility to	*PDX1*, ASP76ASN	[rs137852783]
Diabetes mellitus, Type Ii, susceptibility to	*PDX1*, GLN59LEU	[rs137852784]
Diabetes mellitus, Type Ii, susceptibility to	*PDX1*, 3-BP INS, 243CCG	–
Diabetes mellitus, Type Ii, susceptibility to	*PDX1*, CYS18ARG	[rs137852785]
Diabetes mellitus, Type Ii, susceptibility to	*PDX1*, ARG197HIS	[rs137852786]
Reclassified – variant of unknown significance	*PDX1*, GLU224LYS	[rs137852787]
Pancreatic agenesis 1 Diabetes mellitus, Type Ii, susceptibility to, included	*PDX1*, GLU164ASP	[rs80356661]
Pancreatic agenesis 1	*PDX1*, GLU178LYS	[rs80356662]
Pancreatic agenesis 1	*PDX1*, GLU178GLY	[rs387906777]

dbSNP The Single Nucleotide Polymorphism Database

Pedigree analyses demonstrated diabetes mellitus type 2 in our patient’s mother and some maternal relatives.

### Follow-up and treatment

On the 28th day, he was diagnosed as having hyperglycemia (serum glucose level was 496 mg/dL) by blood examination and treatment with regular daily insulin injections (1.0 unit/kg per day) was started immediately. The insulin infusion rates were regularly titrated according to pre-prandial blood sugar levels; the type of lactation was breast with fortified formula. Weight and height trend in relation to initiation and follow-up of insulin injection is presented in Table 3. Glibenclamide was added later (at 47 days of age) and stopped at the age of 60 days. However, the baby continued to get regular isophane insulin (NPH).

**Table 3:** Patient’s weight and height trend in relation to initiation and follow-up of insulin injection

Index	Birthday	2 months and 22 days	4 months and 4 days	6 months and 4 days
Weight (g)	1800	3000	4000	5000
Height (cm)	46	50	55	65

Other treatment operations included infant oxygen hood (oxyhood), nasal continuous positive airway pressure (nCPAP), broad-spectrum antibiotics prescription, granulocyte colony-stimulating factor (G-CSF), human intravenous immunoglobulin (IVIG), fresh frozen plasma (FFP), cryoprecipitate, surgery of duodenum atresia (at 28 days of age), phototherapy, ursodeoxycholic acid (UDCA), fat-soluble vitamins prescription, and total parenteral nutrition (TPN) for 65 days. He was discharged at 65-days old against medical advice, transferred to the city of residence, and was supervised by a gynecologist.

A summary of relevant past interventions and their outcomes is presented in Table 1.

## Discussion and conclusions

Here, we report a syndromic case of NDM with mutation in *PDX1* gene. This was a novel homozygous mutation, p.Phe167Val, is located in the gene on chromosome 13 (c.499 T>G). His mother had suffered from gestational diabetes and some second-degree relatives had diabetes mellitus type 2. There was no evidence of agenesis of the pancreas. Various single gene and chromosomal abnormalities have been identified that cause different manifestations of NDM [4].

NDM is permanent in nearly half of the patients and may be caused by mutations affecting genes that play a critical role in cell development, cell survival, or cell function. Recently, monogenic causes were recognized in 50% of cases of permanent insulin-dependent diabetes occurring before the age of 6 months [5]. Syndromic NDM is most commonly a result of mutations in *FOXP3*, *EIF2AK3*, *PTF1A GLIS3*, *NEUROD1*, *HNF1B*, immunodysregulation polyendocrinopathy enteropathy X-linked (IPEX) syndrome, and *PDX1*. *PDX1* also termed *IPF1* encodes insulin promoter factor1 with additional features of agenesis of the pancreas [4].

*PDX1* has been reported as a main factor in pancreas development and function [6, 7]. Mutations in *PDX1* may be involved in several disorders, including agenesis of the pancreas or congenital pancreatic hypoplasia (*PAGEN1*; Online Mendelian Inheritance in Man 260370) and diabetes mellitus (for example, Online Mendelian Inheritance in Man 222100; MODY, Type 1, Online Mendelian Inheritance in Man 125850) [8]. Clearly, if a patient presents with features consistent with a syndrome, testing for the relevant mutation should be carried out first. However, if negative, testing for the most prevalent causes in descending order should be carried out. With the exception of IPEX and *HNF1B*-related diabetes, the syndromic causes of NDM are autosomal recessive, carrying a 25% recurrence risk in subsequent children. A correct diagnosis that allows the proper treatment to be selected should lead to better glucose control and improved health in the long term. Testing of other family members may also be indicated to determine whether they are at risk for diabetes. According to studies, the same defect that causes PNDM or TNDM can be present in parents or other first-degree relatives and be diagnosed as type 1 diabetes, monogenic diabetes of youth, or type 2 diabetes mellitus as subsequently detailed. Current evidence suggests that the mutation is likely to be pathogenic. The first cases (three patients from two families) with biallelic *PDX1* mutations that had complete pancreatic agenesis were found by Nicolino *et al*. (2010) [9]. Later, De Franco *et al.* (2013) identified three cases with permanent neonatal diabetes (2.9%). One proband and his affected brother were compound heterozygotes for a frameshift and a novel missense mutation (p.A34fsX191; c.98dupC and p.P87L; c.260C>T). The other two probands were homozygous for novel *PDX1* missense mutations (p.A152G; c.455C>G and p.R176Q; c.527G>A) [5].

This study reported a novel mutation related to NDM with mutation in *PDX1* gene. Mutation in this gene is rare but detection of it is vital. Correctly distinguishing monogenic NDM from type 1 diabetes presenting in infancy critically impacts treatment decisions, surveillance of complications and associated conditions, and has important genetic implications for siblings and offspring of affected individuals.

## Conclusion

The *PDX1* gene in mutation screening for syndromic NDM is introduced as a genetic diagnosis even in the absence of pancreas appearances.

## Additional file

Additional file 1: Figures S1 and S2: Molecular genetic laboratory reports. (ZIP 2090 kb)

## Data Availability

All data generated or analyzed during this study are included in this published article. Data sets will be available if requested.

## Abbreviations

Hb: Hemoglobin
MODY: Maturity-onset diabetes of the young
NDM: Neonatal diabetes mellitus
*PDX1*: Pancreatic and duodenal homeobox 1
PLT: Platelet
PNDM: Permanent neonatal diabetes mellitus
TNDM: Transient neonatal diabetes mellitus
