# Supplementary figures and images for: Iranian neonatal diabetes mellitus due to mutation in PDX1 gene: a case report
Source: J Med Case Rep. 2019 Aug 1;13:258. doi: 10.1186/s13256-019-2149-x (PMC6670147; doi:10.1186/s13256-019-2149-x)

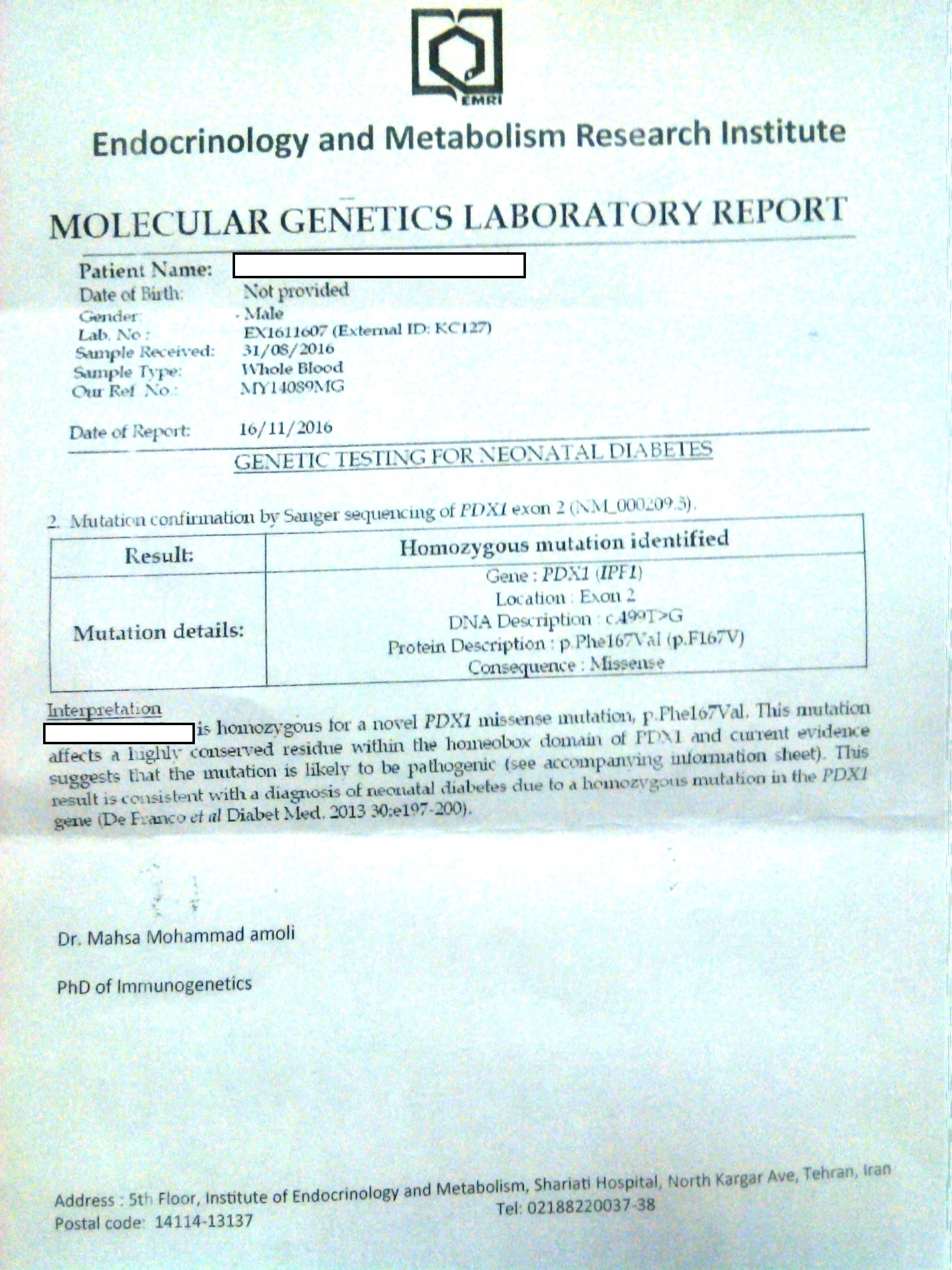

Supplement: Supplementary file 1 — Figures S1 and S2: Molecular genetic laboratory reports. (ZIP 2090 kb) [file 13256_2019_2149_MOESM1_ESM.zip › 13256_2019_2149_MOESM1_ESM/Figure S1.jpg]

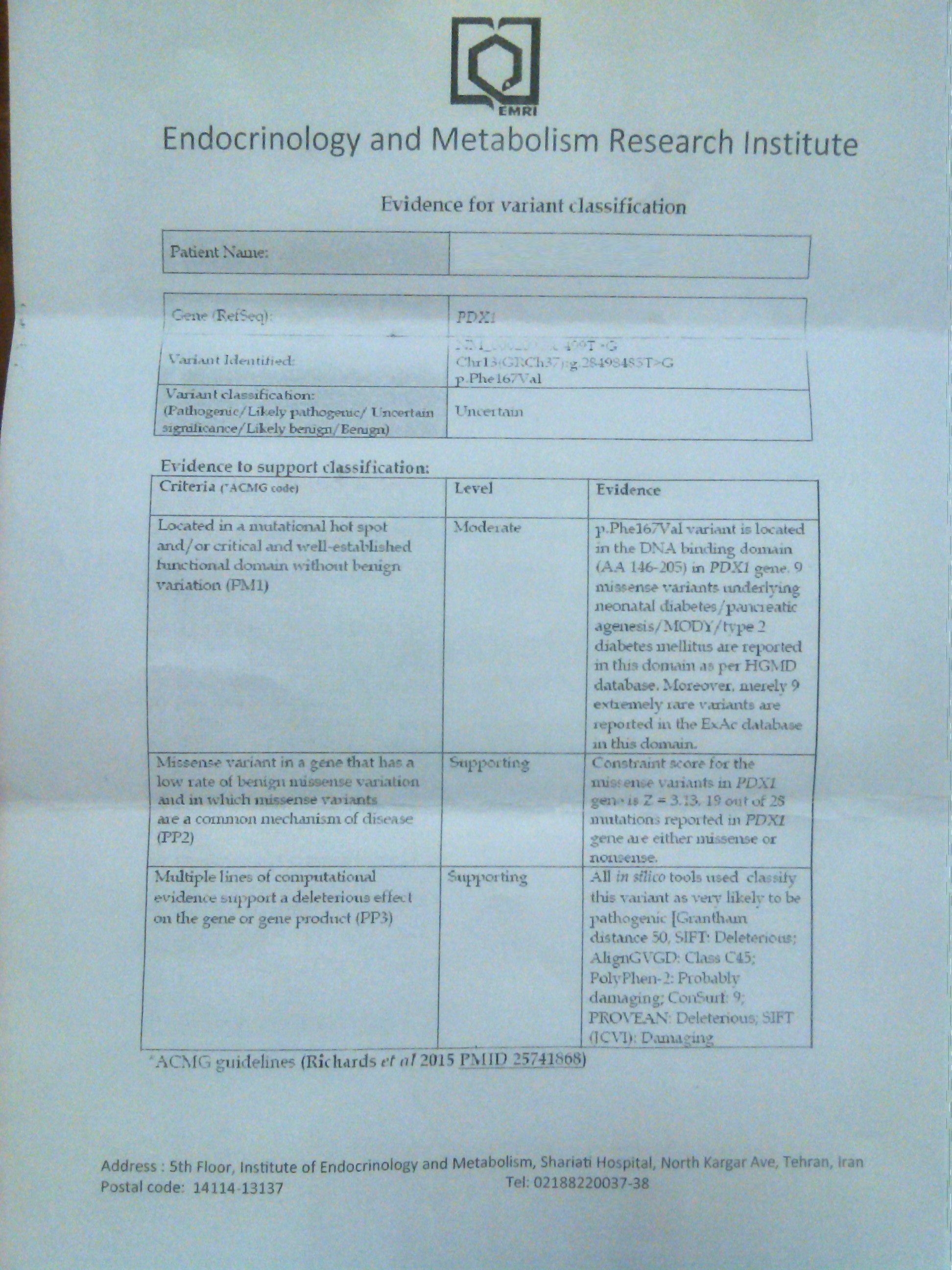

Supplement: Supplementary file 1 — Figures S1 and S2: Molecular genetic laboratory reports. (ZIP 2090 kb) [file 13256_2019_2149_MOESM1_ESM.zip › 13256_2019_2149_MOESM1_ESM/Figure S2.jpg]
